# Prevention of Recurrent Acute Otitis Media in Children Through the Use of *Lactobacillus salivarius* PS7, a Target-Specific Probiotic Strain

**DOI:** 10.3390/nu11020376

**Published:** 2019-02-12

**Authors:** Nivia Cárdenas, Virginia Martín, Rebeca Arroyo, Mario López, Marta Carrera, Carlos Badiola, Esther Jiménez, Juan M. Rodríguez

**Affiliations:** 1 Department of Nutrition and Food Science, Complutense University of Madrid, 28040 Madrid, Spain; niviacu@yahoo.com (N.C.); vmartinmerino@gmail.com (V.M.); rebecaa@vet.ucm.es (R.A.); esther.jimenez@probisearch.com (E.J.); 2 Centro de Salud Bermeo, Tonpoi Kalea, s/n, 48370 Bermeo, Bizkaia, Spain; mario.lopezmateo@osakidetza.eus; 3 Centro de Salud Silvano, Av. de Machupichu, 58, 28043 Madrid, Spain; marta.carrera@salud.madrid.org; 4 Laboratorios Casen Recordati S.L., Vía de las Dos Castillas, 33, 28224 Pozuelo de Alarcón, Madrid, Spain; cjbadiola@casenrecordati.com

**Keywords:** *Lactobacillus salivarius*, otitis, probiotic, bacteriocin, prevention

## Abstract

Acute otitis media (AOM) is one of the most common bacterial infections in children. Empiric antibiotherapy leads to increasing antimicrobial resistance rates among otopathogens and may impair the correct development of the microbiota in early life. In this context, probiotics seem to be an attractive approach for preventing recurrent AOM (rAOM) through the restoration of the middle ear and nasopharyngeal microbiota. The aim of this study was the selection of a probiotic strain (*Lactobacillus salivarius* PS7), specifically tailored for its antagonism against otopathogens. Since *L. salivarius* PS7 was safe and displayed a strong antimicrobial activity against otopathogens, its efficacy in preventing rAOM was assessed in a trial involving 61 children suffering from rAOM. Children consumed daily ~1 × 10^9^ CFU of *L. salivarius* PS7, and the number of AOM episodes were registered and compared with that observed in the previous 6 and 12 months. The microbiota of samples collected from the external auditory canal samples was quantitatively and qualitatively assessed. The number of AOM episodes during the intervention period decreased significantly (84%) when compared to that reported during the 6 months period before the probiotic intervention. In conclusion, *L. salivarius* PS7 is a promising strain for the prevention of rAOM in infants and children.

## 1. Introduction

Acute otitis media (AOM) is one of the most common diseases in infancy and childhood, and the leading cause for seeking medical care and for the prescription of antibiotics, both in high-income and low-income countries [1,2]. Approximately 70% of infants experience at least one otitis episode by the age of 2 years, and 20–30% suffer from recurrent AOM (rAOM) [3]. rAOM is a very relevant issue in clinical practice since it causes pain and discomfort in children, causing a strong impact on their families and a relevant economic burden on society [3].

Middle ear and nasopharyngeal colonization with multiple bacterial otopathogens (mainly *Streptococcus pneumoniae*, *Streptococcus pyogenes*, *Haemophilus influenzae* and *Moraxella catarrhalis*) is considered to be the main risk for both AOM and rAOM [4,5]. Recent microbiome studies have revealed that *Alloiococcus otitidis* may also play a relevant role in the pathogenesis of otitis media [6,7], while other species frequently isolated from the middle ear fluid of children experiencing this condition include *Staphylococcus aureus*, *Staphylococcus epidermidis*, *Pseudomonas aeruginosa* and Escherichia coli [8].

In practice, eradicating or decreasing the concentration of such species through empiric systemic and topical antibiotherapy is usually considered as the main (and, often, unique) approach for the treatment of rAOM and, also, for providing prophylaxis for this condition [9]. However, a routine antibiotherapy may have negative consequences. First of all, it drives the selection of resistant otopathogens [9,10]. Although rAOM episodes occurring within one month from the completion of an antibiotic therapy may be the result of either a relapse or a new infection, antibiotic pressure seems to be essential for selecting the causal agent(s) in both circumstances [11,12]; in fact, the incidence of antibiotic-resistant *S. pneumoniae* and *H. influenzae* strains in nasopharyngeal samples is higher among children with rAOM than among healthy controls [13,14]. In addition, otopathogens, such as *H. influenzae, S. pneumoniae, M. catarrhalis* and *A. otitidis,* form polymicrobial biofilms within the middle ear [15,16,17], and bacteria within these structures have an increased antibiotic resistance [18]. Recently, it was shown that *A. otitidis* promoted *H. influenzae* survival in mixed biofilms by decreasing its antibiotic susceptibility and enhancing its growth under adverse conditions [17]. A Cochrane review [2] aimed to assess the effects of antibiotics for children with AOM revealed that, although they may be useful in children under two years of age with bilateral AOM and otorrhea, their global effect on health outcomes associated to this condition is limited. The same review encouraged the weighing of the benefits of antibiotics against possible harms, including adverse events (such as vomiting, diarrhea or rashes), and suggested that clinical management should provide a limited role for antibiotics, and that a search for new preventive and treatment strategies should be stimulated.

The collateral damage that antibiotics exert on the host’s health by eliminating prominent (but sensitive) members of the microbiota must also be taken into account. Our relationship with our microbiota is especially important during the first years of life, when the microbiome is still forming and any strong disturbance can have short, medium and long-term consequences for homeostasis and health [19,20,21,22,23,24]. The microbiome of the middle ear, ear canal and nasopharynx of healthy children with no history of AOM seems to be characterized by the presence of potentially protective commensal bacteria and the absence or low abundance of classic otopathogens [25].

In this context, probiotics seem to be an attractive approach for preventing rAOM through the restoration of the middle ear and nasopharyngeal microbiota. The lack of specificity of the probiotics used for this target may be one of the main reasons for the limited and contradictory results obtained so far [25,26]. Therefore, the aim of this study was the characterization of a probiotic strain specifically selected for its antagonism against otopathogens. In addition, other potentially properties related to its probiotic potential and safety were investigated, including the assessment of its acute and repeated-doses oral toxicity in a rat model. Finally, the efficacy of the selected strain (*Lactobacillus salivarius* PS7) in preventing rAOM in infants and children was assessed in a pilot clinical trial.

## 2. Materials and Methods

### 2.1. Isolation and Identification of the Strain

Strain PS7 was isolated in a de Man, Rogosa, and Sharpe (MRS, Oxoid, Basingstoke, UK) agar plate within the framework of a previous study to evaluate the bacterial diversity of milk from healthy women. Initially, the identification of the strain was performed by PCR amplification and the sequencing of the 16S rRNA gene using the primers pbl16 (5′-AGAGTTTGATCCTGGCTCAG-3′) and mbl16 (5′-GGCTGCTGGCACGTAGTTAG-3′) [27]. The identification was confirmed by Matrix Assisted Laser Desorption Ionization-Time of Flight (MALDI-TOF) mass spectrometry using a Vitek-MS™ instrument (BioMérieux, Marcy l’Etoile, France) [28]. The strain could be differentiated from other *L. salivarius* strains of our own collection by genotyping using randomly amplified polymorphic DNA (RAPD) analyses, as previously described [29].

### 2.2. Survival after In Vitro Exposure to Saliva and Gastrointestinal-Like Conditions

The survival of the strain to conditions resembling those found in the human digestive tract (saliva, human stomach and small intestine) was assessed in the in vitro system described by Marteau et al. [30], with the modifications reported by Martín et al. [31]. For this purpose, the strain was vehiculated in UHT-treated milk (25 mL) at a concentration of 10^9^ CFU/mL. The values of the pH curve in the stomach-like compartment were those recommended by Conway et al. [32]. Different fractions were taken at 20, 40, 60, and 80 min from this compartment, and exposed for 120 min to a solution with a composition similar to that of human duodenal juice [30]. The survival rate of the strain was determined by culturing the samples on MRS agar plates, which were incubated at 37 °C for 48 h.

### 2.3. Adhesion to Caco-2/HT-29 Cells

The ability of the strain to adhere to HT-29 and Caco-2 cells was evaluated as described by Coconnier et al. [33] with the modifications reported by Martín et al. [31]. HT-29 and Caco-2 were cultured to confluence in 2 mL of DMEM medium (PAA, Linz, Austria) containing 25 mM of glucose, 1 mM of sodium pyruvate and supplemented with 10% heat-inactivated foetal calf serum, 2 mM of L-glutamine and 1% of a non-essential amino acid preparation. At day 10 after the confluence, 1 mL of the medium was replaced with 1 mL of DMEM containing 10^8^ CFU/mL of the PS7 strain. The adherence was measured as the number of lactobacilli adhering to the cells in 20 random microscopic fields. The assay was performed by triplicate.

### 2.4. Production of Riboflavin, Folate and Cyanocobalamin

The riboflavin, folate and cyanocobalamin production by strain PS7 was determined using the microbiological assays described by Juarez del Valle et al. [34], Laiño et al. [35], and Horwitz [36], respectively. *Lactobacillus rhamnosus* ATCC 7469, *L. rhamnosus* NCIMB 10463 and 7469, and *Lactobacillus delbrueckii* B_12_ were used as the indicator organism for the biosynthesis of the respective vitamins. The riboflavin production in the riboflavin-free medium was confirmed by an HPLC analysis following a procedure described previously [34].

### 2.5. Antimicrobial Activity of Strain PS7 against AOM-Related Pathogens

Initially, an overlay method was used as previously described [37] to determine the ability of strain PS7 to inhibit the growth of a spectrum of bacterial strains previously isolated from clinical cases of AOM (own collection of the Complutense University of Madrid) including: *A. otitidis* MP02, *S. pneumoniae* MP07, *S. pyogenes* MP03, *Enterococcus faecalis* MP64, *S. aureus* MP29, *S. epidermidis* MP33, *H. influenzae* MP04, *M. catarrhalis* MP08, *P. aeruginosa* MP24 and *E. coli* MP69. Brain Heart Infusion (BHI, Oxoid, Basingstoke, UK), Columbia Nalidixic Acid (CNA, Biomerieux, Marcy-l’Étoile, France) or Trypticase Soy (TSA, Oxoid) agar plates (depending on the indicator strain) were overlaid with bacterial indicators, incubated at 37 °C for 48 h, and they were examined for zones of inhibition around the PS7 streaks.

### 2.6. Production of Specific Antimicrobials (Bacteriocins, Lactic Acid, Acetic Acid, Hydrogen Peroxide) by Strain PS7

Bacteriocin production was assayed using an agar diffusion method as described by Dodd et al. [38] and modified by Martín et al. [31], using the Gram-positive strains employed for the overlay method as indicator bacteria. Since, at that stage, it was already known that strain PS7 belongs to the *Lactobacillus salivarius* species, the strain was tested by PCR for the presence of the structural genes encoding salivaricins. More specifically, the primer couples used in this study were: (i) SalB-for (5′-TGATAAGAAAGAATTGGCACATATAATTG-3′) and SalB-rev (5′-TCTGTTTAACTACAAATATTTTGATTTGAATG-3′) for salivaricin B [39], and (ii) Abp118A-for (5′-AAACGTGGTCCTAACTGTGTAGG-3′) and Abp118B-rev (5′-AACGGCAACTTGTAAAACCACCAG-3′) for bacteriocin Abp-118 [40]. The PCR reactions were carried out as indicated in the respective articles.

L-and D-lactic acid and acetic acid production by the strain PS7 was quantified in MRS cultures using enzymatic kits (Roche Diagnostics, Mannheim, Germany), as previously reported [31]. The assays were performed in triplicate and the values were expressed as the mean ± SD. The pH values of the supernatants were also measured. Finally, hydrogen peroxide production by the strain PS7 was assayed using the procedures described by Song et al. [41] and by Yap and Gilliland [42]. *L. johnsonii* La1 was used as a positive control in both assays.

### 2.7. Coagregation Assays

The ability of the strain to aggregate with cells of the otitis-related strains cited above was investigated following the procedure of Reid et al. [43], as adapted by Younes et al. [44].

### 2.8. Co-Culture Studies

Broth co-cultures of strain PS7 and some of the otitis-related strains cited above were performed in a BHI broth, since it was observed that this medium allowed most of their growth. The tubes were initially inoculated at a concentration of ~1 × 10^8^ CFU/mL for each of the bacterial strains (PS7 and the corresponding otitis-related strain) and incubated overnight at 37 °C in aerobic conditions. BHI monocultures of each of the strains used in these assays were performed as control cultures. After incubation, samples of all the co-cultures and monocultures were seeded onto MRS, CNA, TSA and BHI agar plates for a selective enumeration based on the ability of the strains to growth and to display differential colony morphologies when inoculated on such media.

### 2.9. Sensitivity to Antibiotics

The sensitivity of the strain PS7 to antibiotics was tested using the lactic acid bacteria susceptibility test medium (LSM) [45] and the microtiter VetMIC plates for lactic acid bacteria (National Veterinary Institute of Sweden, Uppsala, Sweden), as described previously [46]. Parallel, minimum inhibitory concentrations (MICs) were also determined by the E-test (AB BIODISK, Solna, Sweden) following the instructions of the manufacturer. The results were compared to the cut-off levels proposed by the European Food Safety Authority [47].

### 2.10. Formation of Biogenic Amines and Degradation of Mucin

The capacity of the strain PS7 to synthesize biogenic amines (tyramine, histamine, putrescine and cadaverine) from their respective precursor amino acids (tyrosine, histidine, ornithine and lysine; Sigma-Aldrich) was evaluated using the method described by Bover-Cid and Holzapfel [48]. The potential of the strain to degrade gastric mucine (HGM; Sigma, St. Louis, MO, USA) was evaluated in vitro as indicated by Zhou et al. [49].

### 2.11. Acute and Repeated Dose (4-Weeks) Oral Toxicity Studies in a Rat Model

Wistar male and female rats (Charles River Inc., Marget, Kent, UK) were used to study the acute and repeated dose (4-weeks) oral toxicity of the strain PS7 in a rat model. The acclimatization, housing and management (including feeding) of the rats was performed as previously described [50]. The rats were 56-days old at the initiation of treatment. Acute (limit test) and repeated dose (4 weeks) studies were conducted in accordance with the European Union guidelines (EC Council Regulation No. 440) and authorized by the Ethical Committee on Animal Research of the Complutense University of Madrid (protocol 270111).

In the acute (limit test) study, 24 rats (12 males, 12 females) were distributed into two groups of 6 males and 6 females each. After an overnight fasting, each rat received skim milk (500 µL) orally (control group or Group 1), or a single oral dose of 1 × 10^10^ CFU of *L. salivarius* PS7 dissolved in 500 µL of skim milk (treated group or Group 2). The doses of the test and control products were administered by gavage. At the end of a 14-day observation period, the rats were weighed, euthanized by CO_2_ inhalation, exsanguinated, and necropsied.

The repeated dose (4 weeks) (limit test) study was conducted in 48 rats (24 males, 24 females) divided in four groups of 6 males and 6 females each (control group or Group 3; treated group or Group 4; satellite control group or Group 5; and satellite treated group or Group 6). Rats either received a daily oral dose of skim milk (Groups 3 and 5) or of 1 × 10^9^ CFU of *L. salivarius* PS7 dissolved in 500 µL of skim milk (Groups 4 and 6) over 4 weeks. All rats of Groups 3 and 4 were deprived of food for 18 h, weighed, euthanized by CO_2_ inhalation, exsanguinated, and necropsied on day 29. All animals of the satellite groups (Groups 5 and 6) were kept for a further 14-day period without treatment to detect delayed occurrence, persistence or recovery from potential toxic effects. All rats from Groups 5 and 6 were deprived of food for 18 h, weighed, euthanized by CO_2_ inhalation, exsanguinated, and necropsied on day 42.

The behavior and clinical observations, blood biochemistry and haematology analysis, organ weight ratios and histopathological analysis were conducted as described previously [50]. The bacterial translocation to the blood, liver or spleen, and the total liver glutathione (GSH) concentration was evaluated following the methods described by Lara-Villoslada et al. [51].

### 2.12. Pilot Clinical Trial: Prevention of rAOM in Children

In this prospective pilot clinical assay, 64 children (aged 10 months to 6 years) with a previous history of rAOM were recruited between September 2012 and April 2015. The primary outcome variables were the occurrence and duration of the AOM episodes. The secondary end point was the frequency of an otitis-related pathogen carriage in the external auditory canal. The inclusion criteria were at least four episodes of AOM during the preceding 12 months or at least three episodes during the preceding 6 months [52]. The exclusion criteria included chronic medication, chronic illnesses, lip or palatal cleft, programmed tympanostomy or adenoidectomy during the study, and lactose intolerance or cow’s milk protein allergy (because of the excipient used to administer the strain). Those children who had undergone tympanostomy or adenoidectomy during the preceding 6 months were also excluded. The data on the occurrence of AOM episodes from a similar population (age, gender) of otitis-prone children (*n* = 60), who were attended by the same pediatrician but did not receive the tested strain, were used for comparison purposes. Written informed consent was obtained from the parents or legal tutors in accordance with the Declaration of Helsinki. The protocol was approved by the Ethical Committee on Clinical Research of Hospital Universitario Clínico San Carlos (Madrid, Spain), under protocol B12/262.

During the 6-month intervention period, the recruited children consumed daily ~1 × 10^9^ CFU of *L. salivarius* PS7. The parents recorded whether the child had or had not received the daily dose. Compliance was expressed as the percentage of days in which the child received the dose. The use of antibiotics (except for the treatment of an AOM episode) or other probiotics was not allowed during the study. A physical examination was performed by a pediatrician for each suspected AOM episode. AOM was diagnosed according to defined clinical criteria [52,53], including evidence of middle ear effusion, inflammation of the tympanic membrane and any other sign of an acute infection (fever, ear ache, otorrhoea, etc.). The number of AOM episodes and the duration of each episode were also recorded and compared to the same data obtained in the 6 months preceding the probiotic treatment.

Samples from the external auditory canal were collected with a sterile swab at the baseline and at the end (6 months) of the study and submitted for a bacterial analysis following the guidelines recommended by the Spanish Society of Infectious Diseases and Clinical Microbiology [53]. Microbial isolates were identified by a MALDI-TOF analysis as previously reported.

### 2.13. Statistical Analysis

All the quantitative assays included in this study were performed at least in triplicate. The quantitative data were expressed as the mean and standard deviation (SD). When not normally distributed, the data were presented as the median and interquartile range (IQR). For the murine assays, the data were expressed as the means ± standard error of the mean (SEM) of the determinations. The differences between the control and treated groups were evaluated with a one-way analysis of variance (ANOVA) followed by Dunnett’s test. A Wilcoxon signed rank test was used to compare paired microbiological data before and after the probiotic intake and the χ^2^ test was used to find differences in the detection frequencies of the bacterial species in the external auditory canal samples. For all the comparisons, differences were considered significant at *p* < 0.05. The statistical analyses were conducted using R (version 3.0.2, R-project) software [54].

## 3. Results

### 3.1. Identification and In Vitro Characterization of the Strain

Strain PS7 was identified as a member of the *L. salivarius* species (from now, *L. salivarius* PS7). A RAPD genotyping analysis showed that the profile of this strain was different to those of other *L. salivarius* strains from our own collection. Subsequently, the strain was deposited at the Spanish Type Culture Collection (CECT; Burjassot, Spain) under accession number CECT 9422.

In relation to the survival of *L. salivarius* PS7 after exposition to conditions resembling those found in the human digestive tract, an exposure to a saliva-like solution had no deleterious effect on the strain while the survival rate after a transit through the stomach- and small intestine-like compartments was at approximately 52% of the population initially inoculated.

The strain produced neither folates nor cyanocobalamin under the assayed conditions. In contrast, it was able to produce riboflavin at a total concentration of ~200 ng/mL (intracellular riboflavin: 165.00 ± 0.52 ng/mL; extracellular riboflavin: 34.74 ± 3.06 ng/mL).

*L. salivarius* PS7 showed a high ability to adhere to both Caco-2 and HT-29 cells. The mean ± SD of the number of adhered lactobacilli in 20 random microscopic fields was 697.1 ± 297.6 and 251.7 ± 82.3, respectively.

**Figure 1:**
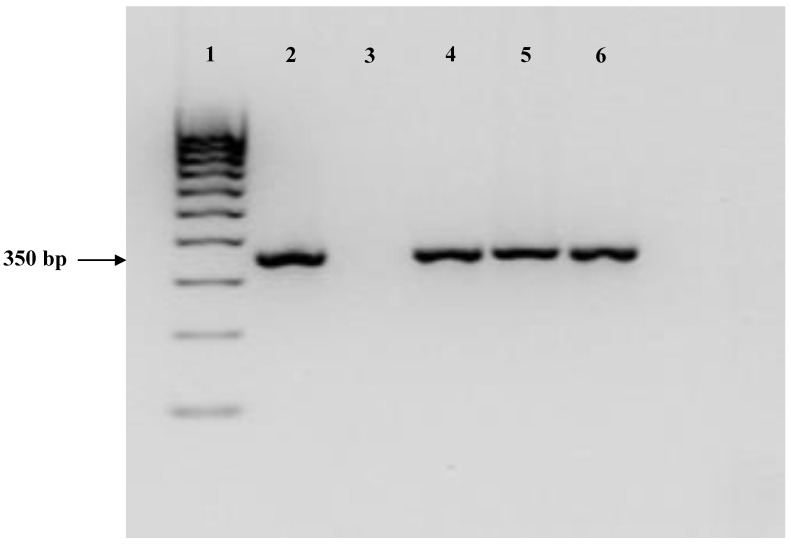
PCR assay for the detection of the bacteriocin Abp118 structural gene. Lane 1: marker (Hyperladder^TM^ 100 bp, BIOLINE, London, UK). Lane 2: positive control. Lane 3: negative control. Lanes 4, 5 and 6: *L. salivarius* PS7.

*L. salivarius* PS7 showed a clear antimicrobial activity (inhibition zone >2 mm around the streak) against most of the otitis-related indicator organisms used in this study (Table 1). Subsequently, and to try to elucidate the compound(s) responsible for the antimicrobial activity, the strain was screened for production of bacteriocins, organic acids (D-and L-lactic acid, acetic acid) and hydrogen peroxide. The strain displayed bacteriocin activity against the Gram-positive strains included as indicator organisms in this study (Table 1). A PCR analysis for structural genes of known salivaricins revealed that this strain produced the bacteriocin Abp-118 (both structural genes were identical) (Figure 1). *L. salivarius* PS7 produced a high concentration of L-lactic acid in the MRS broth while it did not produce the D-lactic acid isomer (Table 2). A significant concentration of acetic acid could also be detected in the culture supernatants of the strain (0.68 ± 0.17 mg/mL). This strain did not produce hydrogen peroxide.

*L. salivarius* PS7 showed a high potential for co-aggregating with bacterial strains involved in AOM cases, particularly with those belonging to the genera *Streptococcus*, *Alloiococcus*, *Enterococcus* and *Staphylococcus*. In addition, most of the otitis-related Gram-positive pathogens could not be detected, or their concentrations decreased notably after their overnight co-culture with the strain PS7 in the BHI broth (Table 1).

The antibiotic sensitivity assays showed that all the MIC values were below the cut-off values recommended by EFSA for all antibiotics, with the exception of kanamycin (MIC in this study: 128 µg/mL; EFSA cut-off value: 64 µg/mL) (Table 3). However, recent reports suggest that *L. salivarius* may be intrinsically resistant to kanamycin, and this aspect will be discussed below. Finally, *L. salivarius* PS7 was able neither to degrade gastric mucin in vitro nor to produce biogenic amines.

### 3.2. Acute and Repeated Dose (4-Weeks) oral Toxicity Studies in a Rat Model

All animals survived both oral toxicity trials. The development of the treated animals during the experimental periods corresponded to their species and age. At no time point of the experimental period were there any significant differences in body weight or body weight gain among the groups treated with *L. salivarius* PS7 (including the satellite ones) in comparison to the control groups. No abnormal clinical signs, behavioural changes, body weight changes, haematological and clinical chemistry parameters, macroscopic or histological findings, or organ weight changes were observed. There were no statistical differences in body weights among the groups. Similarly, no statistically significant differences in body weight gain, or in food and water consumption were observed between the groups. 

No significant differences in the liver GSH concentration were observed between the control and treated groups (9.67 ± 1.42 vs. 9.71 ± 1.56 mmol/g, *p* > 0.1), and lactobacilli could not be isolated from the blood, liver or spleen of the treated rats.

### 3.3. Pilot Clinical Trial: Prevention of AOM in Children

**Figure 2:**
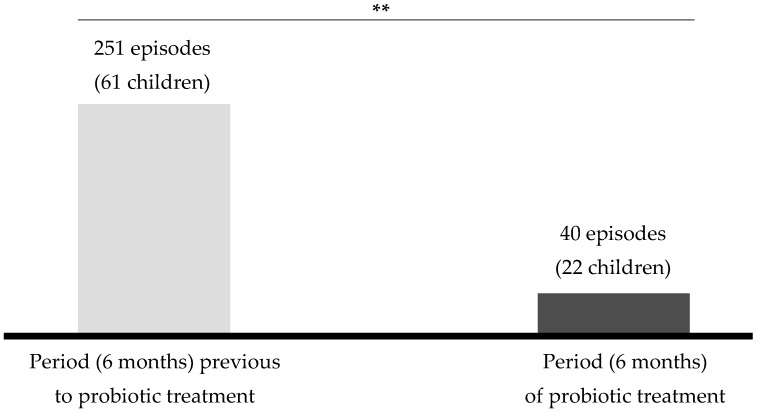
Comparison between the number of AOM episodes reported during the 6 months of the probiotic treatment and those reported during the previous 6 months. **, statistically significant change (*p* < 0.05; χ^2^ test).

A total of 64 children who fulfilled the inclusion criteria were enrolled and received the probiotic treatment (Table 4). Three children (~4.6%) dropped out (one due to antibiotic intake; one due to tympanostomy; one due to a previously unadvertized allergy to cow’s milk protein); thus, 61 children completed the study. Compliance during the study was very high (≥ 96%). Twenty-two out of the 61 recruited children (36%) suffered at least one episode of AOM (median [25Q–75Q]), with a median duration of 4 days (Table 4). In contrast, ~70% of the children with rAOM who were attended by the same pediatrician in the same period but did not receive the probiotic strain suffered at least one AOM episode with a median duration of 6 days. The number of AOM episodes during the 6-month intervention period decreased by 84% when compared to the number registered in the 6 months that preceded the intervention (Figure 2). Although any child suffering an AOM episode received an antibiotic treatment, the number of antimicrobial treatments decreased by ~60% in the children receiving the probiotic strain with respect to the other otitis-prone children treated by the same pediatrician. 

Globally, the microbial density in the external auditory canal decreased notably along the intervention period, from ≥3.5 log_10_ CFU at the baseline (all the cultures were positive at this sampling time) to ≤ 2 log_10_ CFU at the end of the intervention (with 22% of the cultures being negative). At time 0, *M. catharralis*, coagulase-negative staphylococci and *A. otitidis* were the dominant bacteria in swabs taken from the external auditory canal of the recruited children, while coagulase-negative staphylococci and viridans streptococci dominated after 6 months of the probiotic treatment (Table 5). *A. otitidis, H. haemolyticus, H. influenzae*, *M. catharralis*, *Neisseria* spp, *P. aeruginosa*, *S. aureus*, *S. pneumoniae* and *S. pyogenes*, which were relatively common in the samples obtained at time 0, were not detected or their frequency of detection decreased notably at the end of the intervention (Table 5).

## 4. Discussion

In the present study, the probiotic potential of *L. salivarius* PS7 was characterized by using a wide variety of in vitro and in vivo assays, including its ability to antagonize otopathogens, its toxicological assessment in rats, and a pilot clinical trial for the initial evaluation of its efficacy for preventing rAOM in infants and children. The characterization scheme of this strain followed the guidelines for the evaluation of probiotics provided by FAO and WHO [55].

Both the survival rate (52%) of *L. salivarius* PS7 when exposed to conditions similar to those found in the digestive tract and its capacity for adhesion to intestinal cells were similar or higher than those found for commercial probiotic strains in previous studies using the same in vitro models [31,56]. These properties indicate that a high concentration of the strain can reach the pharynx and the gut mucosal surfaces and adhere to them. The adhesion ability of this strain may allow the competitive exclusion of pathogenic bacteria [57]. While its presence in the pharynx may facilitate its direct interaction with otopathogens, its presence in the gut might also have positive consequences. It has been shown that exposure (after swallowing) of the gut to upper respiratory pathogens involved in otitis media provides a potent modulation of the immune responses in the middle ear [58]. The potential ear-related immunomodulatory properties of *L. salivarius* PS7 were not addressed in this work but deserve future research.

In order to select a strain for the prevention of otitis, its ability to exert antagonism against otopathogens seems an essential property. The results of the in vitro assays to evaluate the antimicrobial activity of *L. salivarius* PS7 against a wide variety of strains and species previously isolated from clinical rAOM cases confirmed that it was a suitable candidate for such a target. All the otitis-related indicator strains were inhibited when the overlay method was used and, among them, all the Gram-positive strains were sensitive to the bacteriocin-like compound produced by *L. salivarius* PS7. The PCR analyses revealed that *L. salivarius* PS7 produces Abp-118. This is a potent broad-spectrum class II bacteriocin, which was originally described in *L. salivarius* UCC118 [59]. A previous study demonstrated that Abp-118 is produced in vivo and that it was the primary mediator conferring protection against infection by *Listeria monocytogenes* in mice [60]. The lack of inhibition of a few otopathogens (*E. faecalis* MP64) in the co-culture assay may be due to the fact that Abp-118 is not expressed when the bacteriocinogenic strain grows in a BHI medium. In addition, *L. salivarius* PS7 produced high amounts of L-lacic acid in the MRS cultures, and this is in agreement with the results obtained with other *L. salivarius* strains in previous studies [31,56]. Smaller concentrations of acetic acid were also present in the MRS cultures. The fact that *L. salivarius* PS7 cells were able to co-aggregate with most of the otopathogenic strains tested in this work may facilitate the exposure of otopathogens to the antimicrobial substances produced by *L. salivarius* PS7 [44]. Recently, it was shown that, to exert antimicrobial activity in mucosal surfaces, physical contact between lactobacilli and pathogens may be required [61]. Interestingly, the presence of lactobacilli in the nasopharynx has been associated with a reduced risk of respiratory conditions in children [62]. Unfortunately, the presence of *Lactobacillus* in ear or nasopharynx samples was not tested in this study.

Although *L. salivarius* is a species included among those with the “qualified presumption of safety” (QPS) status [63], *L. salivarius* PS7 was submitted to a thorough safety assessment. In relation to its pattern of antibiotic sensitivity, all the MIC values were below the cut-off values recommended by EFSA [47], with the exception of kanamycin. A resistance of lactobacilli to kanamycin is a common finding among *L. salivarius* strains [64,65,66] due to the lack of a transport system for this antibiotic. This type of intrinsic resistance does not represent a human health risk [47]. In a previous study, the MIC value of *L. salivarius* CECT 5713 (a commercial probiotic strain) for kanamycin (128 µg/mL) [46] was the same as the one obtained with *L. salivarius* PS7 in this study. The analysis of the genome of *L. salivarius* CECT 5713 revealed the absence of transmissible genes involved in kanamycin resistance. Moreover, *L. salivarius* PS7 did not produce biogenic amines and did not show any ability for mucin degradation. These results are also similar to those reported for other *L. salivarius* strains using the same assays [31,56]. An acute and repeated-dose oral toxicity assessment in rats showed that *L. salivarius* PS7 was completely safe in this animal model when administrated in doses ranging from 9 to 10 log_10_ CFU daily. No significant differences in the liver GSH concentration were observed between the control and treated groups, indicating that the treatment did not cause oxidative stress to rats. This result is consistent with the absence of bacteremia since no lactobacilli could be isolated from the blood, liver or spleen of the rats, indicating that the tested strains do not cause either a local or a systemic infection in rats. These findings are in agreement with those previously reported for another *L. salivarius* strain also isolated from human milk [51].

Because of the wide antimicrobial activity of *L. salivarius* PS7 against otopathogens and its safety, a pilot clinical trial was carried out to assess its efficacy in preventing rAOM in infants and children. Oral intakes of *L. salivarius* PS7 over 6 months led to a statistically significant reduction (84%) in the number of episodes of AOM in comparison to those observed in the same population during the 6 months preceding the probiotic intervention. Such a percentage is higher than that observed in previous studies after oral or nasal administration of other probiotic strains, such as *Lactobacillus rhamnosus* GG and LC705, *Bifidobacterium breve* 99 and *Propionibacterium freudenreichii* JS [67,68], and *Streptococcus salivarius* 24SMB or *Streptococcus oralis* 89a [69,70]. When AOM occurred, the median duration of AOM episodes was 4 days, which is lower than the value obtained in previous probiotic trials (5.6 days) [68].

The external ear canal may act as a bacterial reservoir for the same otopathogens usually found in the middle ear during AOM [17,25,71]. Therefore, ear canal samples were analyzed before and after the probiotic intake period. The probiotic treatment led to a statistically significant decrease in the number of ear canal samples that were positive for bacterial growth and in the bacterial density of the positive samples (Table 5). The frequency of detection of potential otopathogens (*A.otitidis, Haemophilus haemolyticus, H. influenzae*, *M. catharralis*, *Neisseria* spp., *P. aeruginosa, S. aureus, S. pneumoniae* and *S. pyogenes*) in the samples also decreased notably after the trial (Table 5). The only bacterial groups whose detection frequency remained similar before and after the probiotic treatment were coagulase-negative staphylococci and viridans streptococci. This change in the composition of the ear canal microbiota seems to be closely associated with the decrease in the number of AOM episodes. In previous studies, otopathogens were more frequently detected in children with otitis than in healthy controls [10,72,73]. In fact, a failure to erradicate otopathogens from the ear and/or nasoparhyngeal surfaces has been viewed as an explanation for rAOM episodes after treatments with antibiotics [4] or with generic non-otitis-targeted probiotics [68,74]. A lack of specificity may explain the conflicting evidence on the effectiveness of probiotics in preventing AOM [75,76]. In contrast, the presence of coagulase-negative staphylococci and viridans streptococci seems to be a feature of a healthy ear and nasopharynx [13,77].

We acknowledge that the clinical trial performed in this study must be considered as a preliminary “proof of concept” trial since it faces some design limitations (e.g., lack of a placebo group and randomization). However, the results have shown that, while antibiotics may still play a relevant role in treatment of AOM, a target-specific probiotic strain may have a preventive role, significantly reducing the number of episodes in children with rAOM and the number of antibiotic doses required to treat them. This is a relevant finding within the context both of the present “antibiotic resistance crisis” and of the steady rise in autoimmune diseases associated with host microbiota disturbances. Additional work is in progress in order to initiate a well-designed multicenter clinical trial to further confirm the results obtained in this work.

## Figures and Tables

**Table 1:** Antimicrobial activity of *L. salivarius* PS7 against bacterial strains isolated from clinical cases of AOM, as assessed by different assays.

Indicator Strain	Overlay Method (cm)	Well Diffusion Assay (cm)		Co-Cultures (CFU/mL)	
		Non-Adjusted pH	pH 6.2	Initial Load	Final Load
*S. pneumoniae* MP07	3.6	1.1	1.1	7.40	Nd ^a^
*S. pyogenes* MP03	2.0	1.2	0.7	7.74	Nd ^a^
*S. aureus* MP29	1.6	1.3	1.2	7.52	4.81 ^a^
*S. epidermidis* MP33	2.3	1.1	1.0	7.53	5.90 ^a^
*A. otitidis* MP02	1.0	1.2	1.2	–	–
*E. faecalis* MP64	0.5	0.4	0.4	8.02	8.78 ^b^
*H. influenzae* MP04	2.2	–	–	–	–
*M. catarrhalis* MP08	2.1	–	–	–	–
*P. aeruginosa* MP24	1.5	–	–	–	–
*E. coli* MP69	1.4	–	–	7.70	5.74 ^a^

^a^*p* < 0.01. ^b^ *p* = 0.29; Nd, no growth detected; – not assayed. AOM: Acute otitis media; CFU: colony-forming unit.

**Table 2:** Final pH and production of organic acids (mg/mL; mean ± SD) in an MRS broth by *L. salivarius* PS7.

pH	L–Lactic Acid	D-Lactic Acid	Acetic Acid
4.01± 0.04	10.29 ± 0.70	Nd	0.68 ± 0.17

Nd, not detectable.

**Table 3:** Minimum inhibitory concentrations (MICs) and cut-off values (μg/mL) of a variety of antibiotics for *L. salivarius* PS7.

Antibiotics	Cut-Off Values *	MIC (*L. salivarius* PS7)
Ampicillin	4	0.5
Clindamycin	4	0.5
Chloramphenicol	4	2
Erythromycin	1	0.25
Streptomycin	64	32
Gentamicin	16	2
Kanamycin	64	128
Tetracyclin	8	2
Vancomycin	n.r.	>128
Linezolid	2	1
Penicillin	1	0.25

* EFSA (2018), except for linezolid and penicillin (Klare et al., 2007). n.r.: not required.

**Table 4:** Main demographic characteristics of the infants and children recruited in the pilot trial (*n* = 61), and main outcomes of the study.

Characteristic	Sex	*n*	Mean ± SD or Median *	*p*-Value
Age (Years) and Gender			3.31 ± 1.7	
<3 years (*n* = 30)	Males	16		
	Females	14		
≥3 years (*n* = 31)	Males	15		
	Females	16		
Inclusion months:				
September		7		
October		5		
November		6		
December		7		
January		6		
February		10		
March		9		
April		6		
May		3		
June		2		
History of AOM episodes/child				
Preceding 6 months			4 (3–4) *	<0.001 ^¥^
Preceding 12 months			5 (5–6) *	<0.001 ^¥^
During the study			0 (0–1) *	

*n*, number of children; * Median (25Q–75Q); ^¥^ Wilcoxon rank sum test paired comparison.

**Table 5:** Results of the microbiological analysis of the samples from the external auditory canal taken at day 0 before the probiotic intervention and at the end of the 6-month treatment period.

	Time 0	After 6 Months	*p*-Value
Number of positive samples (bacterial growth)	61	17	< 0.001 ^¥^
Median log_10_ CFU (per swab)	4 (3.5–5)	2 (1.5–2)	0.012 *
Number of samples with the presence of:			
*Actinomyces europaeus*	2	0	
*Alloiococcus otitidis*	12	2	
Coagulase-negative staphylococci	14	15	
*Haemophilus haemolyticus*	11	1	
*Haemophilus influenza*	7	1	
*Moraxella catharralis*	14	3	
*Neisseria* spp.	6	0	
*Pseudomonas aeruginosa*	4	0	
*Rhodococcus ruber*	2	0	
*Staphylococcus aureus*	11	3	
*Streptococcus pneumoniae*	9	3	
*Streptococcus pyogenes*	10	6	
Group viridans streptococci	9	11	

^¥^ Chi-squared test. * Wilcoxon rank sum test.
